# Development of Protocols for Regeneration and Transformation of Apomitic and Sexual Forms of Dallisgrass (*Paspalum dilatatum* Poir.)

**DOI:** 10.3389/fpls.2021.787549

**Published:** 2022-02-25

**Authors:** Gustavo E. Schrauf, Lisandro Voda, Alicia M. Zelada, Ana María García, Andrea Giordano, Pablo Peralta Roa, Juan Guitian, Juan Rebori, Sergio Ghio, Luciana Couso, Lautaro Castro, Eduardo Musacchio, Pablo Rush, Jutta Nagel, Zeng Yu Wang, Noel Cogan, Germán Spangenberg

**Affiliations:** ^1^Facultad de Agronomía, Universidad de Buenos Aires, Buenos Aires, Argentina; ^2^Criadero “Cultivos del Sur” FAUBA, Buenos Aires, Argentina; ^3^BASF Argentina S.A., Buenos Aires, Argentina; ^4^Laboratorio de Agrobiotecnología, Departamento de Fisiología, Biología Molecular y Celular, Facultad de Ciencias Exactas y Naturales, Universidad de Buenos Aires, Buenos Aires, Argentina; ^5^Instituto de Biodiversidad y Biología Experimental y Aplicada, Consejo Nacional de Investigaciones Científicas y Técnicas-Universidad de Buenos Aires, Buenos Aires, Argentina; ^6^Agriculture Victoria, AgriBio, Centre for AgriBioscience, Bundoora, VIC, Australia; ^7^School of Applied Systems Biology, La Trobe University, Bundoora, VIC, Australia; ^8^Agriculture Victoria, Hamilton, VIC, Australia

**Keywords:** tissue culture, totipotency, gene technology, selectable marker, molecular analysis, dd-PCR

## Abstract

*Paspalum dilatatum* (common name dallisgrass), a productive C4 grass native to South America, is an important pasture grass found throughout the temperate warm regions of the world. It is characterized by its tolerance to frost and water stress and a higher forage quality than other C4 forage grasses. *P. dilatatum* includes tetraploid (2*n* = 40), sexual, and pentaploid (2*n* = 50) apomictic forms, but is predominantly cultivated in an apomictic monoculture, which implies a high risk that biotic and abiotic stresses could seriously affect the grass productivity. The obtention of reproducible and efficient protocols of regeneration and transformation are valuable tools to obtain genetic modified grasses with improved agronomics traits. In this review, we present the current regeneration and transformation methods of both apomictic and sexual cultivars of *P. dilatatum*, discuss their strengths and limitations, and focus on the perspectives of genetic modification for producing new generation of forages. The advances in this area of research lead us to consider *Paspalum dilatatum* as a model species for the molecular improvement of C4 perennial forage species.

## Introduction

*Paspalum dilatatum* (common name dallisgrass), a productive C4 grass native of South America, is an important pasture grass throughout the temperate warm regions of the world (Holt, 1956; Hutton and Nelson, 1964). *P. dilatatum* exhibits higher forage quality and higher tolerance to frost than other C4 forage grasses (Hacker et al., 1974; Robinson et al., 1988; Davies and Forde, 1991). *P. dilatatum* includes tetraploid (2*n* = 40) sexual forms and pentaploid (2*n* = 50) apomictic forms. Sexual forms are mainly distributed in the temperate humid zones of Argentina, Uruguay, and part of southern Brazil, whereas, apomictic forms are also spread to the southeastern United States, Australia, New Zealand, and tropical Africa (Pizarro, 2000). *P. dilatatum* is a species with C4 photosynthetic metabolism, spring–summer–autumn growth, very plastic, so it adapts to various edaphic and environmental conditions of temperatures and humidity, and it is resistant to defoliation and has a great regrowth capacity (Acosta et al., 1994). Genetic improvement is one of the most effective ways to increase productivity of forages and consequently livestock production (beef, milk, and wool) (Wang and Ge, 2006).

The School of Agriculture of the University of Buenos Aires developed two cultivars by conventional breeding procedures: Relincho, apomictic 2*n* = 50 (INaSe, 2003) and Primo, sexual (self-pollinated) 2*n* = 50 (INaSe, 2013). Relincho has high forage quality and high field establishment, but its seed production is very low because of its susceptibility to *Claviceps paspali*. Primo showed resistance to *C. paspali*, higher seed production and lower forage quality than Relincho. Primo was obtained by means of the backcross methodology, with introgression of *P. urvillei*, a source of resistance to *C. paspali* (Schrauf et al., 2003).

The apomictic reproductive system, which characterizes most of the dallisgrass accessions, reduces the possibility of combining genetic information. Earlier Reusch (1961) raised the need to resort to new breeding methodologies to improve apomictic *P. dilatatum.* Bashaw and Hoff (1962), Burton and Jackson (1962), and Owen (1979) generated mutations through radiation, but no variants of agronomic importance were obtained. Also, an attempt was made to explore somaclonal variation, although tissue or protoplast culture generated some variations, in some cases, it was only transient and generally of little agronomic value (Akashi and Adachi, 1992; Davies and Cohen, 1992; Burson and Tischler, 1993). Burson and Tischler (1993) considered that the application of biotechnological techniques would bring the solution to the problems of dallisgrass.

Considering the genetic complexity and the associated difficulties encountered by conventional breeding methods, transgenic and genome editing approaches offer many alternative and effective strategies to improve forages (Wang and Ge, 2006; Wolabu Tezera et al., 2020). The implementation of transgenic technologies in forage species can improve agricultural profitability, achieving higher productivity, better use of resources such as soil nutrients, water or light, and a reduction in environmental impact. Obtaining new varieties with better forage quality, resistant to pests and diseases, more efficient in acquiring nutrients, and/or with greater tolerance to abiotic stress can be achieved by introducing new high-impact traits in forage through improvement programs (Giraldo et al., 2019). The development of methods to obtain transgenic plants make it possible to expand genetic variability through the access and potential use of genes present in other species. In the recent years, the amount of gene sequences available in databases, and also the sequences of transcriptional regulatory regions or promoters, have increased rapidly due to the development of massive genome sequencing techniques. A step for biotechnological approaches is a robust regeneration protocol since for the use of genetic technologies, such as transformation and genome editing, so that a high efficiency of plant regeneration, is essential.

According to McDougall (2011), for each transgenic event that is selected and commercially released, on average, more than 6,000 are discarded. This means that it is essential to obtain robust and efficient protocols for regeneration and transformation to access genes of relevant agronomic value for forage species.

## Regeneration Protocols

The establishment of stable, efficient *in vitro* regeneration systems is a prerequisite for biotechnology and molecular breeding applications. Factors influencing regeneration are varied, ranging from origin of explant, culture conditions, hormonal effects, and genotype (Neelakandan and Wang, 2012). For the initiation of regenerable callus cultures in *P. dilatatum*, the use of different explants has been explored, for example, mature seed, mature or immature embryos, leaf bases, shoot apices, and immature inflorescences. In *P. dilatatum* and *Paspalum notatum*, plants have been achieved from different explants: inflorescences (Bovo and Mroginski, 1986; Akashi and Adachi, 1992; Burson and Tischler, 1993), ovaries (Bovo and Quarín, 1983), anthers (Bovo et al., 1985), and immature embryos (Bovo and Mroginski, 1989). Using mature embryos or caryopsis to initiate callus culture was the most practical and convenient method. Immature embryos or inflorescences sometimes give good culture response, but isolating these explants is more time-consuming and difficult. Prior to sterilization of caryopses, the glumes and glumeles were mechanically removed. The optimum conditions to sterilize explants were a concentration of 3% of sodium hypochlorite and a 4-min immersion time (Voda, 2021). When glumes and glumeles were not removed, the use of 15 min immersion of seeds in 50% (v/v) sulfuric acid was successful for the sterilization of the explants, but a lower proportion of explants were induced (Giordano et al., 2014b). When the explants came from greenhouse plants, they were easier to sterilize than those from the field. The caryopses produced in spring and early summer showed a greater callus induction than those produced in late summer.

**FIGURE 1:**
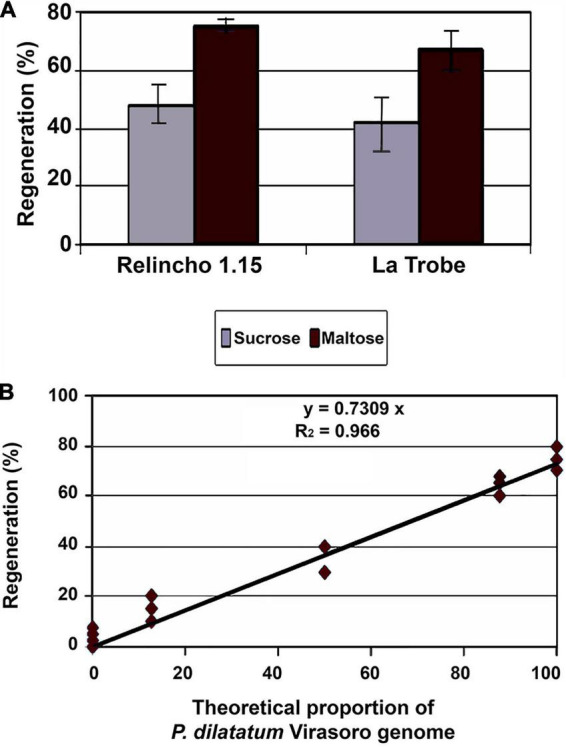
Effects of type of carbon source and genetic variation upon regeneration. **(A)** Estimated regeneration frequencies through the ratio number of regenerated explants or number of proliferated explants, of two apomictic genetic materials (Relincho 1.15 and La Trobe) grown in induction and proliferation media with sucrose or with maltose (differences between media within genotypes were significant through the *G*-test (*p* < 0.05). Source: Schrauf (2009). **(B)** Regeneration of EC from progeny derived from interspecific crosses between *P. dilatatum* (Virasoro) and *P. urvillei.* Linear association between theoretical proportion of Virasoro and regeneration. Source: Schrauf (2009).

The MS (Murashige and Skoog, 1962)-based media supplemented with 2,4-dichlorophenoxyacetic acid (2,4-D) were used for callus induction and maintenance. Later on, 2,4-D was replaced by kinetin for the regeneration process. These media were described in detail by Spangenberg (1995) and Forster and Spangenberg (1999). The replacement of sucrose by maltose as a carbon source in these media improved calli embryogenesis in apomictic forms (Figure 1A), and similar results were found in different species (Ganesan and Jayabalan, 2005; Sharma et al., 2005).

Although the totipotency depends upon the effect of genotypes, a protocol is robust if it is applicable to a wide range of genotypes. Several apomictic accessions including Relincho cultivar were analyzed for the induction, proliferation of embryogenic calli (EC), and regeneration. Osmopriming pretreatments to dormant seeds of *Paspalum* accessions with polyethylene glycol (PEG) (Schrauf et al., 1995) significantly improved embryogenic callus induction (Table 1). Apomictic genotypes without seed dormancy showed high value of regeneration, and this value was increased using explants from regenerant plants of the same genotype (Table 2). These results were replicated in other genotypes and can be explained by epigenetic effects of tissue culture (Schrauf et al., 2000).

**TABLE 1:** Proportion of induced embryogenic calli (EC) (number of induced calli/Number of explants), from seeds (pretreated or not pretreated with PEG) from different genetic sources.

Genetic sources			Callus induction (%)	
Species	Accessions	Genotypes	without PEG	with PEG
*Paspalum dilatatum*	Relincho	Apomitic genotype	69^b^	81^a^
*Paspalum dilatatum*	Virasoro	Sexual population	56^b^	69^a^
*Paspalum dilatatum*	Las Chilcas	Apomitic population	21^b^	50^a^
*Paspalum dilatatum*	Paysandú	Apomitic population	14^b^	54^a^
*Paspalum dilatatum*	Cucullú	Apomitic population	5^b^	23^a^
*Paspalum dilatatum*	Campomar	Apomitic population	0^b^	6^a^
*Paspalum dilatatum*	La Trobe	Apomitic population	0^b^	8^a^
*Paspalum dilatatum*	Covas	Apomitic genotype	61^b^	83^a^
*Paspalum dilatatum*	Müller	Apomitic genotype	48^a^	53^a^
*Paspalum dilatatum*	Alonso	Apomitic genotype	37^b^	66^a^
*Paspalum dilatatum*	Villar	Apomitic genotype	17^b^	61^a^
*Paspalum urvillei*	Fauba	Sexual population	17^a^	14^a^

*Different letters indicate significant differences within rows, G-test, (p < 0.05). Source: Schrauf, 2009.*

**TABLE 2:** Efficiency of induction (number of induced calli/number of explants), proliferation (number of proliferated calli/number of induced calli) and regeneration (number of regenerated calli/number of proliferated calli) of explants from the Relincho genotype and its regenerants (R1).

Material	Induction	Proliferation	Regeneration	Final
Relincho (glasshouse)	82.3^a^	85.2^a^	87.3^ab^	61.2^a^
Relincho R1 (1st year)	98.6^b^	90.1^a^	95.5^b^	84.8^b^
Relincho R2 (field)	74.0^a^	85.0^a^	82.5^a^	51.9^a^
Relincho R1 (2nd year)	100^b^	98.7^a^	80.9^a^	79.8^b^

*Different letters indicate significant differences, G-test, (p < 0.05). Source: Schrauf, 2009.*

The tissue culture behavior of sexual sources was also analyzed. Caponio and Quarín (1990) obtained interspecific hybrids between *P. urvillei* (vaseygrass) X *P. dilatatum* biotype Virasoro. Resistance to ergot infections of *P. urvillei* was transferred to several plants in the backcrosses, showing that hybridization between both species provides a new approach to obtain *C. paspali* (ergot) resistance and improve seed production of dallisgrass (Schrauf et al., 2003). It is assumed that the materials from backcrosses (BC2) have 87.5% of the genome of the recurrent parents and the material derived from an F2 50% of both genomes. The Figure 1B shows that the proportion of *P. dilatatum* (Virasoro) genome was associated with greater capacity to regenerate under the analyzed conditions. The coefficient of determination was very high, showing *r*^2^ = 0.96.

For genetic transformation purpose, plant regeneration system has to be highly efficient and reproducible. It is critical to identify and selectively enrich the type of callus that is highly embryogenic. EC and embryogenic cell suspensions (ECSs) can be used as targets for biolistic transformation (Potrykus et al., 1998). Cell suspension culture is initiated by agitating embryogenic callus in liquid medium. Cell suspension cultures are also the only source for obtaining totipotent protoplasts in monocots. However, maintenance of ECSs involves routine subculture that is time-consuming and risk contamination (Wang et al., 1993). As ECSs lose their totipotency capacity when the culture is maintained for a prolonged period of time, subculture requires cryopreservation (Wang et al., 1993). In *P. dilatatum*, regeneration from ECSs showed similar results than EC (Schrauf et al., 2000). But, for practicality, the development of EC was chosen. In case of need to fix a genotype with high regeneration, the maintenance of vegetative *in vitro* tillers was used (Giordano et al., 2014a,b).

## Biolistic Transformation Methods

Although the transformation of numerous forage grasses has been reported, initially only the transient transformation of callus has been reported in *P. dilatatum* (Yuge et al., 1998; Akashi et al., 2002). These protocols probably failed in the use of the selectable marker. Schrauf et al. (2001) reported obtaining transgenic plants expressing selection marker genes, and Giordano et al. (2014a,b) and Peralta Roa et al. (2015) reported the gene silencing and the expression of transgenes with high agronomic value, respectively. All of these events were obtained by biolistic transformation.

**FIGURE 2:**
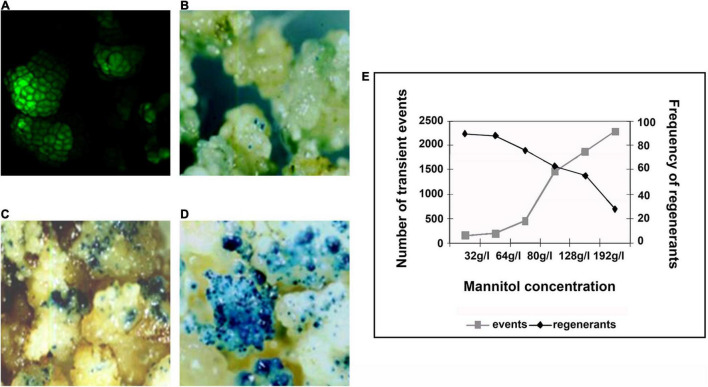
Use of reporter genes for the development of a biolistic transformation protocol. **(A)** Bombarded calli expressing green fluorescent protein (GFP), observed on a confocal microscope. **(B–D)** Bombarded calli expressing GUS osmopretreated with mannitol [**(B)** 32g/l, **(C)** 64g/l, and **(D)** 96g/l]. **(E)** Association between the concentration of mannitol, number of transient events, and frequency of regenerants. Source: Schrauf (2009).

Biolistics, or microprojectile bombardment, can be defined as the introduction of DNA employs high-velocity metal particles into intact cells for stable transformation (Sanford, 1988). Embryogenic cultures, composed mostly of proembryogenic cell clusters and groups of meristematic cells rich in cytoplasm, can be used as suitable targets for biolistic transformation (Spangenberg and Wang, 1998). For the development of biolistic transformation protocol, β-glucuronidase (GUS), and green fluorescent protein (GFP) (Figure 2A) genes were used as reporters. An acceptable transient GUS expression is between 1,000 and 2,000 blue spots per Petri plate (Sanford, 1988; Wang et al., 1988). Five days prior to bombardment, osmotic pretreatments with various concentrations of mannitol (from 32 to 192 g/l) were assayed. Higher concentration of mannitol implied an increase in the number of transient events, but concentrations greater than 96 g/l implied a reduction in the frequency of regenerants (Figures 2B–E).

**FIGURE 3:**
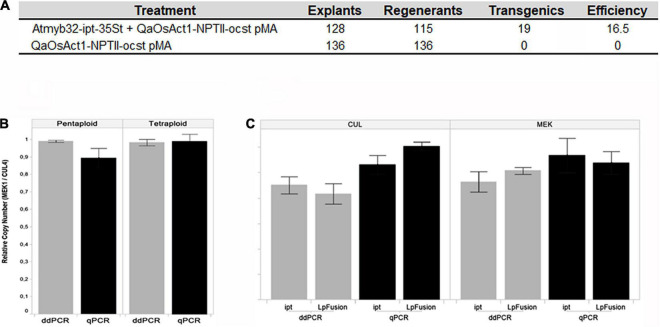
**(A)** Effect of *Atmyb32:ipt* on regeneration. Comparison of the efficiency in obtaining transgenic regenerants, in a medium without selector, with or without the *Atmyb32: ipt* construction. Source: Voda (2021). **(B,C)** Comparison of qPCR and dd-PCR techniques, **(B)** in pentaploid and tetraploid genotypes. Histograms indicate the relationship between two unique copied endogenous genes (*Cul4* and *Mek1*), **(C)** in copy number detection of two transgenes (ipt and *LpFusion*) using two endogenous reference genes (*Cul4* and *Mek1*). Histograms indicate the average of copy number. Vertical lines indicate the standard deviation. Source: Voda (2021).

As frequently only a very small proportion of cells are transformed in most experiments, the chances of recovering transgenic lines without selection are usually low (Miki and McHugh, 2004). Since the choice of the selectable marker gene is key step in the transformation protocol setting, different combinations of selectable marker gene or selector were assayed. The use of *hph* gene with hygromycin did not produce regenerants, but the use of *pHp23 (nptII)* gene with kanamycin produced a large number of escapes, whereas, paramomycin showed the highest efficiency (Schrauf, 2009). Yuge et al. (1998) and Akashi et al. (2002) found similar results using *hph* and hygromycin in *P. dilatatum*. Voda (2021) found that *Atmyb32:ipt* gene can be used as selectable marker, without requiring the use of a selector agent, in the apomictic Relincho cultivar (Figure 3A). Endo et al. (2001) claimed that *ipt* gene is effective as a selectable marker gene for plant transformation in tobacco. *Atmyb32:ipt* can play a similar role as BABY BOOM transcription factor (BBM) to improve the capacity of recalcitrant plant to *in vitro* regeneration *via* tissue culture (Lutz et al., 2015; Jones et al., 2019).

Two biolistic helium-accelerated particle systems were used: BIOMICS-EMBRAPA and PDS-1000/He (Bio-Rad), both showed similar results, but PDS showed a tendency toward higher efficiency and higher frequency of multiple transgene copies (Schrauf, 2009).

## Determination of Transgene Copy Number

Molecular analysis of transgenic plants was undertaken with both PCR and RT-PCR to assess the presence and expression of transgene, respectively. PCR was used to evaluate the variants of the transformation protocols (as for example in Figure 3A). Although the final value is given by the phenotype and agronomic evaluations, the number of copies of the transgene of each event is of crucial importance for deregulation.

Southern blot, real-time qPCR, and digital droplet PCR (dd-PCR) estimated the copy number of transgenes. Both single copy events and multiple copy events were found by Southern analysis (Schrauf, 2009). Typically, events with low transgene copy number are preferred to avoid potential transgene silencing and to integrate the events into improvement programs. Based in our results dd-PCR was identified as the optimal strategy for high accuracy copy number determination (Figures 3B,C; Głowacka et al., 2016; Narancio et al., 2021). Also, Giraldo et al. (2019) found higher sensitivity and repeatability in transgene detection with the dd-PCR technology than with qPCR.

## Perspectives

**FIGURE 4:**
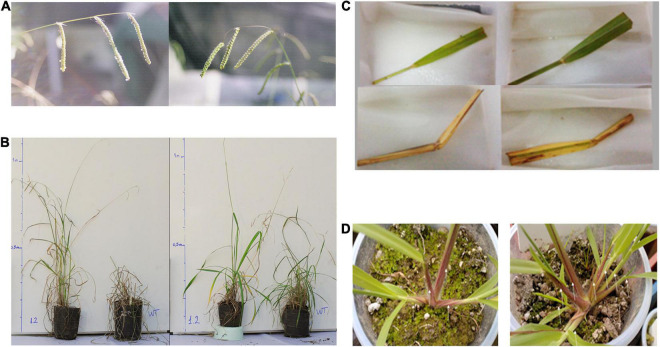
**(A)** Transgenic event with tolerance to biotic stress: Dallisgrass inflorescences 15 days after inoculation with *C. paspali*, (left) control, (right) transgenic plant expressing chitinase (pUbi:Chi5B). Source: (Schrauf et al., 2021). **(B)** Transgenic event with tolerance to abiotic stress: Acquisition of tolerance to salinity through overexpression of a sodium or proton vacuolar antiporter (left) transgenic plant Atnhx1 vs. wild-type plant under saline condition, (right) both under non-saline condition. Source: Schrauf et al. (2020). **(C,D**) Transgenic events of delayed senescence. **(C)** Comparison of senescence of excised leaves of (upper) transgenic Atmyb32:ipt plants with (below) wild-type plants. **(D)** Comparison of initial tillering between (left) wild-type and (right) transgenic Atmyb32:ipt plants. Source: Voda (2021).

**FIGURE 5:**
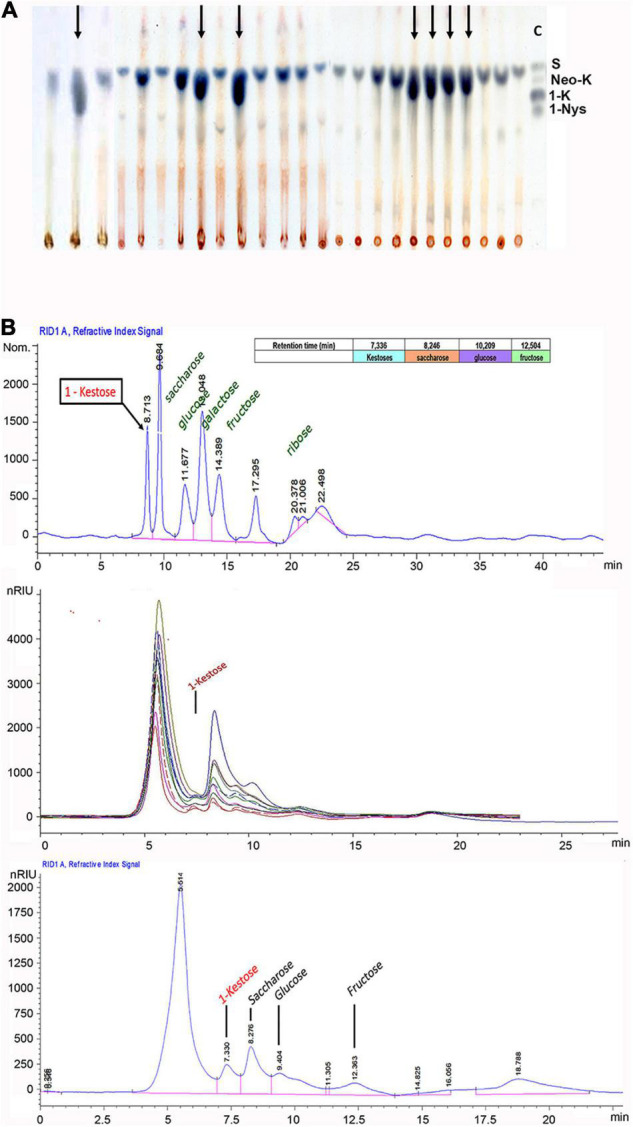
High energy (fructan) transgenic events. **(A)** Thin-layer chromatography of different samples of soluble sugars from qPCR-positive plants of *P. dilatatum*. Black arrows indicate the possible presence of kestoses in the sample. As a control C, S = sucrose, neo-K = neo-kestose, 1-K = 1-kestose, and 1-Nys = 1-nystose standards were used. Source: Peralta Roa et al. (2015). **(B)** HPLC analysis of soluble sugars from qPCR-positive plants. Run of fructose, glucose, ribose, sucrose, saccharose, and kestose standards (upper). Sugar samples of transgenic *P. dilatatum* (medium). Run of a single qPCR-positive sample of *P. dilatatum* (lower). Source: Peralta Roa et al. (2015).

The adjustment of robust regeneration and transformation protocols has allowed obtaining numerous transgenic events with a high-potential agronomic value. Within these events, it is possible to list the following:

(1) Antifungal protein (glucanases, quitinase) under constitutive promoter (pUb:GLU, pUb:GLUApo, pUbi:Chi5B) for the resistance to *Claviceps paspali* (Figure 4A). This trait is the most relevant for the apomictic forms of *P. dilatatum* (Holt, 1956; Hampton, 1984; Schrauf et al., 2021). The entry pathway for *C. paspali* is through the floral style-stigma, and the expression of antifungal peptides under the control of appropriate organ-specific promoters is being explored.
(2) One strategy to increase salinity tolerance is overexpression of the *Arabidopsis thaliana* nhx genes that code for a sodium or proton vacuolar antiporter (Blumwald et al., 2000); the incorporation *via* transgenesis of the Atnhx1 and Atnhx5 genes shows qualitative effects (Figure 4B; Schrauf et al., 2020). Having the *P. dilatatum* genome will allow exploring the overexpression of its own antiporter genes.
(3) The use of *Atmyb32-ipt* will allow at least two applications, as a selectable marker (Figure 3A) and improving forage value through delayed leaf senescence and increase tillering (Figures 4C,D).
(4) Fructan gene integration and expression in *P. dilatatum* (Figures 5A,B) will allow the increase in forage quality, tolerance to abiotic stress, and indirectly mitigating climate change (Valluru and Van den Ende, 2008; Parsons et al., 2011; Rigui et al., 2019).
(5) One strategy to the increase, the forage quality is lignin gene silencing. Lignification of plant cell walls is largely responsible for lowering digestibility of forage tissues. Gene silencing was obtained by expression of a frame-shift mutant of a Cinnamoyl-CoA Reductase (CCR) gene, which was delivered separately to the selectable marker cassette of study by Giordano et al. (2014a,b). Silencing ***via*** genome editing is also analyzed for the near future (Cox et al., 2017).

The effect of each of these transgenes is being explored and for (3), (4), and (5) stacking them have begun.

Perennial forage grasses form the foundation for grassland agriculture and play important roles in environmental protection and are ideal for certain value-added products because they permit multiple harvests without reestablishment (Bingham and Conger, 1995). Because forage production is generally a low-cash-input system, the most economical way to deliver advanced technology to farmers and ranchers is through the genetic improvement of cultivars (Wang et al., 2001).

Biotechnological approaches will have a huge impact to improve forage yield and quality and tolerances to biotic and abiotic stresses. Wang et al. (2001) claimed the need to drastically improve transformation efficiency and thus allow the production of large numbers of transgenic grasses in a relatively short time period. The latter was especially essential for apomictic forms of *P. dilatatum*. The robust protocols obtained allow us to consider *P. dilatatum* as a model species for the molecular improvement of C4 perennial forage species and to study the genetic control of apomixis.

Also, the use of transgenic grasses may provide a cost-effective way of phytoremediation for the control and removal of soil contaminants and can be considered for molecular farming and for ethanol production from lignocellulose feedstocks. However, controversy over genetically modified (GM) crops led to considerable opposition to the cultivation and use of transgenic plants. Probably, technologies, such as intragenic, cisgenic, and genome editing, will reduce the controversy. The controversies and public concerns are not only on the use of GM technology, rather they are also about intellectual property law where biotech companies can play monopoly. Although public concerns might be valid to some extent, research must not slow down on advancing of these promising technologies because such advancements may well lead to yet more powerful technologies in favor of public (Sticklen, 2015).

## Author Contributions

GES, AZ, LV, NC, and PPR wrote the manuscript. GES, AMG, LV, JG, PPR, AG, EM, LCo, JR, and PR provided the data search and information. GES, SG, AZ, JN, LCa, AG, ZYW, NC, and GS conducted research and/or collaborated in data analysis. All authors contributed to the article and approved the submitted version.

## Conflict of Interest

LV was employed by company BASF Argentina SA. The remaining authors declare that the research was conducted in the absence of any commercial or financial relationships that could be construed as a potential conflict of interest. The handling editor declared a shared affiliation with one of the authors GS at the time of review.

## Publisher’s Note

All claims expressed in this article are solely those of the authors and do not necessarily represent those of their affiliated organizations, or those of the publisher, the editors and the reviewers. Any product that may be evaluated in this article, or claim that may be made by its manufacturer, is not guaranteed or endorsed by the publisher.
